# Disease-Promoting Effects of Type I Interferons in Viral, Bacterial, and Coinfections

**DOI:** 10.1089/jir.2014.0227

**Published:** 2015-04-01

**Authors:** Sophia Davidson, Mala K. Maini, Andreas Wack

**Affiliations:** ^1^ Division of Immunoregulation, MRC National Institute for Medical Research, Mill Hill, London, United Kingdom.; ^2^ Division of Infection and Immunity, University College London, London, United Kingdom.

## Abstract

While type I interferons (IFNs) are universally acknowledged for their antiviral and immunostimulatory functions, there is increasing appreciation of the detrimental effects of inappropriate, excessive, or mistimed type I IFN responses in viral and bacterial infections. The underlying mechanisms by which type I IFNs promote susceptibility or severity include direct tissue damage by apoptosis induction or suppression of proliferation in tissue cells, immunopathology due to excessive inflammation, and cell death induced by TRAIL- and Fas-expressing immune cells, as well as immunosuppression through IL-10, IL-27, PD-L1, IL-1Ra, and other regulatory molecules that antagonize the induction or action of IL-1, IL-12, IL-17, IFN-γ, KC, and other effectors of the immune response. Bacterial superinfections following influenza infection are a prominent example of a situation where type I IFNs can misdirect the immune response. This review discusses current understanding of the parameters of signal strength, duration, timing, location, and cellular recipients that determine whether type I IFNs have beneficial or detrimental effects in infection.

## Introduction

**FIG. 1.**
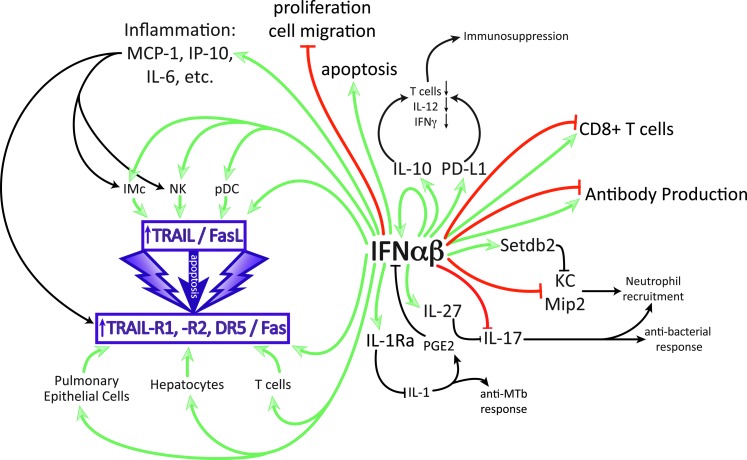
Multiple effects of IFN-αβ on immune and epithelial cells that can contribute to disease in specific infections. Only direct enhancing (*green*) and inhibitory (*red*) effects by IFN-αβ are color coded, secondary effects are in *black*. IFN-αβ can enhance production of IL-10, proinflammatory cytokines, and IL-27 and can block directly or indirectly the cytokines, IL-1, IL-12, IL-17, IFN-γ, and KC. Detrimental effects can be due to the suppression of antibacterial responses (IL-1, IL-17, IFN-γ, and KC) or to overstimulation of inflammation leading to damage by apoptosis of tissue cells or immune suppression by apoptosis of immune cells.

The prototype antiviral cytokine family, type I interferons (IFNs), inhibits replication and spread of a range of viruses both *in vivo* and *in vitro* (Isaacs and Lindenmann 1957; Muller and others 1994; Schneider and others 2014). In addition, type I IFNs can signal to almost every cell type in the body, including immune cells, and thus are potent immunomodulators, ensuring a timely and robust immune response to an invading pathogen. The pluripotency of type I IFNs allows for interaction with the immune system, promoting secretion of specific cytokines such as IL-10 and IL-6 and blocking production or function of others, for example, IL-17, IL-1, and IFN-γ (Fig. 1). Yet, within these extensive and strong effects on the immune response also lies the potential for pathogenesis (Trinchieri 2010).

This review will focus on the detrimental effects type I IFNs can have in infection, where immunomodulatory actions of type I IFNs supersede their protective potential through inappropriate skewing of the host immune response, immunosuppression, or direct damage to the host tissue. For clarity, we assess separately the pathogenic potential of type I IFNs in the context of acute and chronic viral infection, bacterial infection, and finally viral–bacterial coinfection. It should be noted that for most of the type I IFN-mediated effects discussed in this review, the molecular mechanisms are still being investigated and many of these effects may be indirect.

Type I IFNs are a multigene cytokine family that comprises multiple, partially homologous IFN-α subtypes (13 in human and 14 in mouse), a single IFN-β gene, and several other family members (IFN-ω, -ɛ, -δ, and -κ) (Pestka and others 2004). Despite their variety, all members of the type I IFN family exclusively signal through a ubiquitously expressed heterodimeric receptor, the IFN-αβR, encoded by the *Ifnar1* and *Ifnar2* genes. Unlike their other family members, IFN-α and IFN-β are broadly expressed and therefore the most intensely studied. Consequently, the studies mentioned in this review primarily focus on the action of IFN-α and IFN-β.

IFN-αβ acts in both an autocrine and paracrine manner. Engagement of the IFN-αβR upregulates the Janus kinase (JAK)/signal transducers and activators of the transcription (STAT) pathway (Murray 2007). JAK/STAT signaling induces 3 molecules, STAT1, STAT2, and IRF9, to form the trimeric transcription factor, ISGF3, and it is ISGF3 that triggers the transcription of a diverse suite of genes known as IFN-stimulated genes (ISGs) (Ivashkiv and Donlin 2014; Schneider and others 2014).

## Viral Infection

### Acute viral infection: IFN-driven inflammation and tissue damage

Type I IFN inhibits viral replication and spread through the induction of hundreds of genes, appropriately named ISGs, in both infected and noninfected cells. Well-studied ISGs, such as Protein kinase R (PKR) and 2′-5′oligoadenylate (OAS), inhibit the cellular translation machinery or degrade ssRNA to effectively impair virus spread (Sadler and Williams 2008; Schneider and others 2014). Coupled with this induction of antiviral proteins, IFN-αβ also induces secretion of cytokines and chemokines and pathways that allow for clearance of infected cells.

Virus-mediated tissue damage is a hallmark of many acute infections; however, the immune response itself, if left unchecked, can also cause pathology. For example, production of proinflammatory cytokines in high amounts in patients infected with highly pathogenic influenza strains is commonly associated with tissue damage and therefore disease severity (Peiris and others 2004; de Jong and others 2006). Many studies have assessed the contribution of IFN-αβ to this and reported that type I IFN expression in some cases correlates directly and in others inversely with host pathology (Cheung and others 2002; Kobasa and others 2007; Zeng and others 2007; Baskin and others 2009). Cheung and others (2002) recorded IFN-β as one of the first cytokines secreted by avian influenza (H5N1)-infected human macrophages and this preceded induction of other proinflammatory cytokines and chemokines, such as MCP-1, Mip-1β, and IL-12. Conversely, in the same study, lower pathogenicity influenza strains incited a lower IFN-β response, which correlated with diminished transcription of proinflammatory cytokines. Avian influenza H5N1 strains have also been shown to induce dramatic and sustained expression of type I IFNs in infected lung tissue of nonhuman primates and associated with severe necrotizing bronchiolitis and alveolitis (Baskin and others 2009). However, H5N1 was also demonstrated to attenuate the type I IFN response in a polarized human bronchial epithelial cell model (Zeng and others 2007). Furthermore, a study on the highly pathogenic 1918 influenza strain found that higher susceptibility in infected cynomolgus macaques correlated with low type I IFN induction when compared with a lower pathogenicity strain of flu (Kobasa and others 2007).

**FIG. 2.**
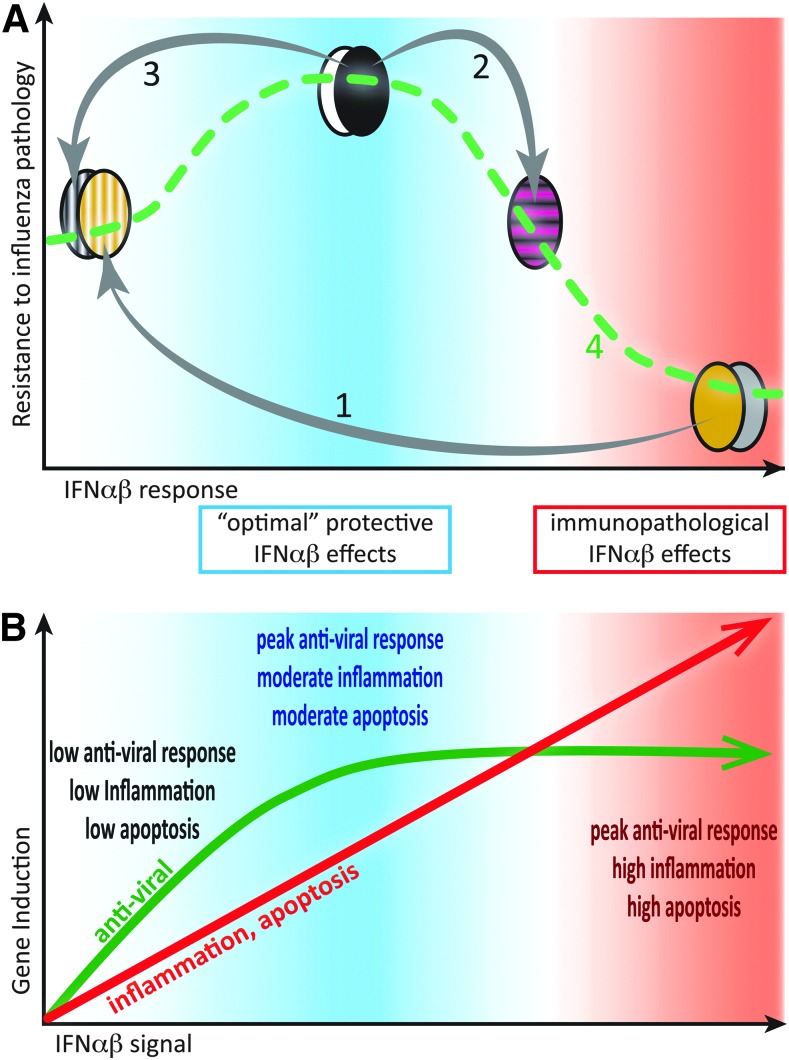
Moderate IFN-αβ responses to infection are protective, while excessive IFN-αβ amounts contribute to immunopathology. **(A)** Bell-shaped curve displaying resistance to influenza severity as a function of IFN-αβ responses, based on experimental findings in Davidson and others (2014) and other studies. High IFN-αβ-expressing mouse strains (eg, 129, DBA, *agouti*, and *gray* symbols) are highly susceptible to influenza, and genetic removal of IFN-αβ from such strains increases resistance (1). Conversely, exogenous addition of IFN-αβ to resistant, low IFN-αβ-expressing mouse strains (eg, C57BL/6, Balb/C, *black*, and *white* symbols) reduces their resistance to influenza (2). However, if the moderate IFN-αβ responses in C57BL/6 mice are genetically removed, influenza resistance is reduced (3). Linking these data points generates a dose–response curve (4) where moderate IFN-αβ responses protect and high IFN-αβ responses are detrimental. **(B)** Induction of antiviral (*green*) versus proinflammatory, antiproliferative, and apoptosis-promoting (*red*) genes as a function of IFN-αβ signal strength based on results and hypotheses, as reviewed in (Piehler and others 2012). We hypothesize that moderate IFN-αβ responses are protective in influenza infections as effective antiviral responses are balanced with moderate inflammation and apoptosis induction. Higher IFN-αβ signals increase inflammation and cell death, but do not further enhance induction of antiviral effectors.

The role of type I IFN in influenza infection remains unclear, and the aforementioned studies are difficult to interpret as they do not allow for the distinction of virus versus host-specific factors that can contribute to pathology. Interestingly, 2 studies that did look specifically at host factors contributing to severe influenza-induced disease associated high IFN-αβ concentrations in the lung during influenza infection with increased disease severity. Boon and others (2009) demonstrated a host-specific correlation between expression of type I IFN and other proinflammatory cytokines with disease severity through the backcrossing of influenza-susceptible (DBA/1) and -resistant (C57BL/6) mouse strains. Moreover, Davidson and others (2014) showed that genetic blockade of IFN-αβ signaling in susceptible 129S7 mice decreased amounts of proinflammatory cytokines and chemokines in the pulmonary environment and concomitantly increased resistance to influenza-induced disease (Fig. 2A). Similarly, infection of *Ifnar1*−/− mice, deficient in the IFN-αβR, with respiratory syncytial virus correlated with lower cytokine secretion in the pulmonary environment compared with wild-type controls, and although this associated with slightly elevated viral loads, it did not impact on host survival (Goritzka and others 2014).

Type I IFNs are known to restrict influenza replication *in vitro* (Isaacs and Lindenmann 1957; Garcia-Sastre and Biron 2006; Trinchieri 2010; Matzinger and others 2013), yet how this translates into a whole organism is less well defined. Pretreatment of mice with type I IFNs 8 h before H5N1 influenza infection did protect against high viral loads and host mortality; however, this was only in mice positive for the potent influenza restricting ISG, Mx1 (Tumpey and others 2007). The murine Mx protein has been demonstrated to be highly effective at restricting influenza, so much so that outbred mice are not natural hosts for influenza, while other species such as chickens or humans who are natural influenza hosts contain Mx homologs that are comparatively less effective, or virus strains have found ways to evade Mx antiviral action in these hosts (Pavlovic and others 1992; Pavlovic and others 1995; Schusser and others 2011).

Pretreatment of ferrets with IFN-α before seasonal influenza infection did assist in virus control and resulted in lower clinical scores compared with mock-treated controls; continuing IFN-α treatment to days 1 and 2 postinfection increased the positive treatment outcome. However, protection was not conferred when ferrets were challenged with avian influenza, instead IFN-α-treated ferrets, in spite of lower viral load in nasal washings, were as susceptible, if not more so, than their vehicle-treated counterparts (Kugel and others 2009). As type I IFN-mediated effects on parameters outside the antiviral response were not investigated, it is unclear whether or not IFN-α treatment affected the proinflammatory cytokine response or drove tissue damage through apoptosis in this model.

Interestingly, treatment with type I IFNs during influenza infection has rarely demonstrated a decrease in disease burden. While Beilharz and others (2007) found moderate oral doses of IFN-α to be somewhat protective during influenza challenge, animals given high doses of IFN-α suffered higher morbidity than placebo controls. Similarly, systemic exogenous IFN-α treatment of an Mx-negative influenza-resistant mouse strain during infection resulted in increased morbidity and mortality rather than protection (Fig. 2A) (Davidson and others 2014).

On balance, exogenous treatment during influenza infection with type I IFNs, or indeed a strong endogenous type I IFN response, appear to exacerbate disease, yet treatment of animals before infection or cells during infection can inhibit viral replication. While on the surface this appears conflicting, we must again return to the concept of type I IFNs as pleiotropic cytokines. Pretreatment of animals with IFN-αβ, if given at the right time, will stimulate the antiviral response in host cells, thereby decreasing virus burden from the start through minimizing the ability of the virus to infect host cells. Treatment of cell lines with type I IFNs during influenza infection can also enhance virus clearance without damage as this is a closed system. However, exogenous addition of type I IFNs in an already inflamed tissue may serve to increase induction of antiviral ISGs [although this may be unlikely given the work by the Schreiber group and others, discussed later, (Fig. 2B)], but will at the same time stimulate the immune system in ways that can exacerbate disease. Protection afforded by IFN-αβ is therefore a function of timing and, more importantly, of concentration (Fig. 2).

Type I IFNs have also been demonstrated to regulate cellular survival and death pathways (Leaman and others 2003; Levin and others 2011). Early studies using acute LCMV infection reported bone marrow aplasia during infection, induced by an IFN-αβ-dependent pathway (Binder and others 1997), although more recent studies in Sendai infection suggest that hemopoiesis, instead of being blocked, can be modified to render developing cells more resistant to virus (Hermesh and others 2010). The death ligand, tumor necrosis factor-related apoptosis-inducing ligand (TRAIL), is upregulated on many cell types in response to type I IFN stimulation (Herold and others 2008; Stary and others 2009; Bernardo and others 2013).

TRAIL, also known as Apo2L, is a member of the TNF superfamily (TNFSF10) of cytokines and acts as either a surface-bound or secreted protein that, upon binding to cells expressing TRAIL receptors with intracellular death domains, can induce apoptosis of target cells in a caspase-dependent manner (Holoch and Griffith 2009). Five TRAIL receptors have been described in both humans and mice; however, it is only TRAIL-R1 and TRAIL-R2 (murine DR4 and DR5, respectively) in humans and only DR5 in mice that contain death domains and therefore have the potential to induce cell death (Schaefer and others 2007; Benedict and Ware 2012). In situations of infection and inflammation, immune cells and other nontransformed cells can upregulate both TRAIL and its death receptor (Fig. 1) (Benedict and Ware 2012). In the context of influenza A virus infection, IFN-αβ-mediated upregulation of TRAIL on inflammatory monocytes (Hogner and others 2013; Davidson and others 2014) and of DR5 on airway epithelial cells resulted in airway epithelial cell death and therefore host morbidity (Davidson and others 2014). Similarly, levels of IFN-αβ-induced FasL (another cell death-inducing molecule) during influenza infection directly correlated with disease severity (Fig. 1) (Fujikura and others 2013).

Influenza is not the only virus to have a complex relationship with type I IFN. A recent study conducted by Wetzel and others (2014) showed that elevated levels of IFN-β in mouse lungs during Sendai virus infection correlated with increased morbidity and mortality. It must be noted that in both Davidson and others and Wetzel and others (2014), the pathogenic potential of IFN-αβ during infection was best revealed when mice were deficient in potent virus-specific inhibiting ISGs: Mx1 for influenza (Pavlovic and others 1995; Tumpey and others 2007) and Ifit2 in Sendai. It is, however, interesting to note that the study by Wetzel and others was performed in C57BL/6 mice, a strain identified as low IFN-αβ producers, while in an earlier study using a high IFN-αβ-producing strain (129 mice), Lopez and others (2006) recorded lower weight loss in *Ifnar1*−/−(129) mice than their wild-type counterparts during Sendai virus infection. It is therefore possible that even in the context of potent antiviral ISGs, high levels of type I IFNs can still drive immunopathology.

The IFN-αβR is ubiquitously expressed, and immunomodulatory effects of type I IFNs are therefore not limited to the innate immune response. Type I IFNs have been shown to contribute to activation of the adaptive immune response through stimulation of dendritic cells (DCs). IFN-αβ can be either a promoting or inhibiting factor in DC development, depending on the stage. It can also enhance cell surface expression of MHC molecules and costimulatory molecules, such as CD80 and CD86, on immature DCs, thereby increasing the ability of DCs to stimulate T cells and promote virus clearance (Ito and others 2001; Montoya and others 2002). However, some viruses such as measles are able to subvert the immune response by reducing the DC pool through stimulation of type I IFN secretion (Hahm and others 2005). Indeed, type I IFNs have been implicated in many forms of virus-mediated immune exhaustion, although this is a feature more characteristic of chronic viral infections.

IFN-αβ can also act directly on CD4^+^ and CD8^+^ T cells in either a promoting or inhibitory manner. For instance, CD8^+^ T cells were shown to expand more strongly in response to soluble antigen when they received an additional signal through their IFN-αβR (Le Bon and others 2006a). A more complex picture was found when bystander CD8^+^ T cell proliferation was studied during viral infection; In this study, a direct IFN-αβR-mediated signal enhanced bystander CD8^+^ T cell expansion early during LCMV infection, but inhibited expansion later in infection (Marshall and others 2011). The notion that the length or timing of the IFN-αβ signal is decisive for the effect on T cell responses was confirmed in West Nile Virus infection where antibody-mediated blockade of IFN-αβ late, but not early, in infection lead to functionally impaired CD8^+^ T cells (Pinto and others 2011). As will be discussed in greater detail in the section on chronic viral infections, even acute LCMV infection leads to increased T cell death that correlated with IFN-αβ levels (Bahl and others 2010). In addition, IFN-αβ has been demonstrated to induce upregulation of CD69 on lymphocytes, which interacted with sphingosine 1-phosphate receptor-1 (S1P1) on the cell surface to inhibit S1P1 chemotactic function, thereby sequestering lymphocytes in lymphoid organs (Shiow and others 2006).

How IFN-αβ affects B cells and the antibody response is unclear. Le Bon and others (2006b) demonstrated that IFN-α is able to stimulate antibody responses and induce isotype switching during an immunization protocol and this was markedly reduced in *in vivo* systems, in which either B cells or CD4^+^ T helper cells were selectively deficient for the IFN-αβR. However, Price and others (2000) reported higher influenza-specific antibody titres in *Ifnar1*−/− mice postinfluenza infection compared with their wild-type counterparts. Through signaling to a wide variety of cell types, IFN-αβ can directly impact the net outcome of the adaptive immune response. In general, this is associated with positive outcomes in acute viral infection; however, since type I IFN is induced in most viral infections, it is possible for viruses to employ IFN-αβ for their own purposes.

A common theme is emerging where type I IFN has the potential to overactivate the immune system during acute viral infection. Mechanisms, which were designed to be protective, such as induction of proinflammatory cytokines or chemokines or activation of apoptosis-inducing pathways to clear virally infected cells, can lead to tissue damage with serious consequences to the host. A delicate balance must therefore be struck where type I IFN signaling must be sufficient to induce an adequate immune response, yet not so overwhelming as to induce immunopathology (Fig. 2A). It appears that nature has accounted for this as expression of antiviral ISGs, such as Mx1, PKR, and OAS2, is acutely sensitive to IFN induction, requiring only picomolar concentrations of type I IFNs, while other ISGs, such as IL-6, CXCL11, and TRAIL, with pathogenic potential require 100-fold higher IFN concentrations (Fig. 2B)(Leaman and others 2003; Coelho and others 2005; Samarajiwa and others 2009; Levin and others 2011; Schoggins and Rice 2011; Thomas and others 2011).

Given that all type I IFNs use the same receptor, it is surprising that there is such variability in ISG induction. In particular, recent evidence indicates that this cannot be explained by differences in ligand binding as all type I IFNs appear to share the same receptor-binding mode [discussed in (Piehler and others 2012)]. Instead, there is a wide spectrum in IFN half-life and ligand affinity to both receptor subunits as well as divergent levels of IFN-αβR expression on specific cell types; these differences translate into differences in gene induction and subsequent biological outcomes. Induction of an antiviral state by type I IFNs is rapidly initiated and maintained over a prolonged time by both high- and low-affinity IFN-αβR ligands (Piehler and others 2000; Kalie and others 2008) and requires binding to only a low number of receptors (Levin and others 2011). Conversely, type I IFN immunomodulatory functions, such as induction of chemokines, inflammation, and regulation of apoptosis, and cell cycle arrest require a prolonged period of signaling as well as high IFN-αβR concentration on the cell surface. Furthermore, effects requiring prolonged IFN-αβ signaling positively correlate to ligand affinity for IFNAR1 and IFNAR2 (Fig. 2B)(Piehler and others 2000; Roisman and others 2005; Kalie and others 2007). Differences in ligand affinity and half-life, as well as cell surface receptor concentration, therefore allow for fine-tuning of the IFN signal at a single-cell level.

To add another layer of type I IFN regulation, upon receptor ligand binding, IFN-αβR is downregulated, thereby allowing for a refractory period. IFN-induced receptor activation leads to ubiquitination of the IFNAR1 subunit (Kumar and others 2004), and through this ubiquitination a conformational change is induced in the receptor, exposing a constitutive endocytic motif, thereby resulting in endocytosis of the active signaling complex (Kumar and others 2007). Failure to downregulate IFN-αβR leads to persistent type I IFN signaling and renders tissue highly susceptible to inflammatory syndromes. Mutant mice unable to stimulate IFNAR1 ubiquitination and therefore receptor downregulation display persistent immune infiltration in inflamed tissues, extensive damage, and impaired tissue regeneration (Bhattacharya and others 2014). How this translates into an acute viral infection scenario is of much interest.

While *Ifnar1*−/− mice have been shown to be susceptible to many virus infections, such as vesicular stomatitis virus (VSV), Semliki Forest virus, vaccinia virus, and lymphocytic choriomeningitis virus (LCMV)(Muller and others 1994), this is not always the case for influenza (Price and others 2000; Mordstein and others 2008; Davidson and others 2014). Increased susceptibility to influenza-induced disease in *Ifnar1*−/− mice has been recorded, yet this has been in the context of high viral doses for mice expressing functional Mx protein (Koerner and others 2007) or in studies where influenza viruses used were capable of causing a systemic infection (Garcia-Sastre and others 1998; Szretter and others 2009).

The variable susceptibility of *Ifnar1*−/− mice to influenza-induced disease caused some confusion in the literature with many studies investigating this; however, differences in influenza strain or dose and use of either *Ifnar1*−/− or *Stat1*−/− mice as models of IFN signaling deficiency on B6, 129, CD1, or mixed mouse backgrounds make comparisons between (and even within) these studies difficult (Garcia-Sastre and others 1998; Durbin and others 2000; Price and others 2000; Mordstein and others 2008; Szretter and others 2009). In particular, it was reasonable in the early studies to assume that in *Stat1*−/− mice type I IFN signaling was specifically ablated. While STAT1 does act downstream of the IFN-αβR, it has more recently been demonstrated to act downstream of the receptors for types II and III IFNs and other cytokines that have wide-ranging effects, such as IL-6, IL-10, and IL-27 (Casanova and others 2012). Thus, the type I IFN-mediated protection suggested to be lost in studies, which have used *Stat1*−/− mice as a model for type I IFN signaling (Garcia-Sastre and others 1998; Durbin and others 2000), is likely a result of a combined effect on a range of cytokines, including type III IFNs.

Since the discovery of type III IFNs (IFN-λ-1, -2, and -3; also known, respectively, as IL-29, IL-28A, and IL-28B in humans, IFN-λ1 being a pseudogene in mice) in 2003 (Kotenko and others 2003; Sheppard and others 2003), much of the controversy surrounding the relationship between type I IFN and influenza has been clarified. Like type I IFNs, IFN-λ is induced during viral infection and signals through the JAK/STAT signaling pathway to activate transcription of ISGs (Sheppard and others 2003; Crotta and others 2013). IFN-λ utilizes a separate receptor complex, and the tissue distribution of the type III IFN receptor is restricted to mucosal surfaces such as gut or airway epithelial cells (Sommereyns and others 2008; Mordstein and others 2010; Durbin and others 2013). Infection of *Ifnar1*−/−, *Ifnlr*−/−, and *Ifnar1−*/−*Ifnlr*−/− double-deficient mice with a range of viruses has demonstrated a level of redundancy between the type I and type III IFN systems. *In vivo* replication of viruses, such as influenza, RSV, and human metapneumovirus (HMPV), were at most only slightly elevated in mice deficient for either IFN-αβR or IFN-λR; however, absence of both receptors caused dramatic loss of virus control and therefore host susceptibility (Mordstein and others 2008, 2010; Crotta and others 2013). Yet, this redundancy does not extend to all viruses; Thogotovirus and Lassa fever virus were also tested within the cited studies, and viral load and host mortality were not altered by loss of IFN-λ signaling.

**FIG. 3.**
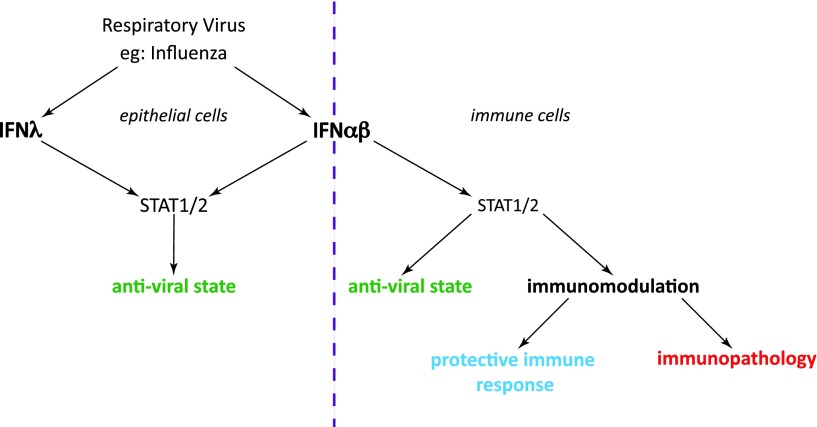
Partial redundancy of types I and III IFNs in infection by influenza and other respiratory viruses. Both IFN-αβ and IFN-λ are induced upon influenza infection. Their effects depend on the distribution of their receptors: Epithelial cells express receptors for both IFN types, and both these IFNs can mediate the induction of the epithelial antiviral response (*left*). Immune cells express only IFN-αβ receptors and can be stimulated or inhibited by type I IFN (*right*). STAT1/2 is involved in signal transduction downstream of both type I and type III receptors. The degree and nature of immune activation determines whether the net result of all combined type I IFN-mediated effects is protective or pathogenic.

Why IFN-λ is protective in some viral infections and not all comes down to virus tissue tropism. Unlike the hepatotropic viruses (Thogotovirus and Lassa fever virus) or indeed other viruses such as LCMV and VSV, which can infect a variety of cell types, influenza, RSV, and HMPV replication are restricted to epithelial cells of the lung (Schickli and others 2005; Collins and Graham 2008; Crotta and others 2013). Thus, type III and type I IFNs are able to act redundantly and restrict spread of viruses whose replication is restricted to cell types, which express both IFN-αβR and IFN-λR, essentially cells at mucosal surfaces (Fig. 3). In contrast, for viruses that can infect cell types not responsive to IFN-λ signaling, including immune cells, neurons, and murine hepatic cells, IFN-λ is not sufficient to protect against virus dissemination.

In the case of influenza and its tissue tropism for airway epithelia, IFN-λ-mediated protection is enough to halt influenza replication and spread in this tissue expressing the IFN-λR, which explains the often mild influenza phenotype of *Ifnar1−*/− mice. An exception to the rule of influenza virus tropism for epithelia is the A/WSN/33 strain, which has the capacity to also infect neurons. Neurons are insensitive to IFN-λ signaling (Sheppard and others 2003) and thus IFN-αβ is uniquely required to combat influenza A/WSN/33 in this cell type. This explains some of the differences between early studies of the role of type I IFN in restricting influenza A/WSN/33 virus (Garcia-Sastre and others 1998) with other studies on type I IFN and influenza (Durbin and others 2000; Price and others 2000; Mordstein and others 2008; Szretter and others 2009; Davidson and others 2014). To summarize, IFN-λ may have evolved to provide a system that allows antiviral factors to be induced in epithelia in the airways, the gut, and other mucosal barriers without triggering the immune system (Fig. 3).

### Chronic viral infection: IFN-mediated shaping of the balance between innate and adaptive immunity

Innate cytokines like type I IFN can be induced in the chronic as well as acute stage of viral infections, potentially playing a major role in shaping immunity and pathogenesis. Studies of chronic LCMV infection in mice have demonstrated both positive and negative regulation of immunity by type I IFNs with effects on T cells directly or through APC (Welsh and others 2012). The pleiotropic effects of type I IFNs on T cells can vary from beneficial (promoting their expansion) to detrimental (inhibiting their proliferation and driving apoptosis), depending on timing and prior antigen experience (Welsh and others 2012; Urban and Welsh 2014). T cell subsets are also differentially affected, for example, regulatory T cells have recently been shown to be relatively resistant to the apoptosis-inducing effects of type I IFN (Che and others 2014). Overall, early in LCMV infection, type I IFN-induced antiviral factors aid the control of viral replication (Muller and others 1994). In contrast, late in infection, blockade of IFN-αβ signaling has been shown to increase LCMV clearance. One study suggested that this disparity in IFN-αβ action was due to type I IFN blocking IFN-γ expression while promoting IL-10 secretion. A parallel study indicated that type I IFN signaling drove expression of programmed cell death 1 ligand (PD-L1) on dendritic cells, antagonized expansion of T cells, B cells, NK cells, and macrophages, and was associated with splenic architecture disorganization (Fig. 1). Importantly, in both these studies, type I IFN-mediated dampening of the immune response impaired CD4^+^ T cells and thereby supported viral persistence (Teijaro and others 2013; Wilson and others 2013).

Type I IFN, either endogenous or therapeutic, has similarly been shown to disable T cell immunity in the major human persistent viral infections, HIV, HBV, and HCV. HIV drives dramatic activation of innate responses upon infection (Stacey and others 2009); prolonged production of IFN-α by pDCs during HIV infection has been proposed to contribute to the persistent immune activation, which is a hallmark of HIV pathogenesis (O'Brien and others 2011; Rajasuriar and others 2013). Progressive disease in Simian immunodeficiency virus (SIV)-infected rhesus macaques is associated with a higher and more sustained IFN-αβ response compared with natural hosts, which do not exhibit disease progression and show lower IFN levels (Mandl and others 2008; Bosinger and others 2009; Jacquelin and others 2009). Furthermore, *ex vivo* analysis of CD8^+^ T cells from HIV-positive individuals found that those categorized with a nonprogressive disease phenotype (ie, resistant to severe virus-induced disease) exhibited lower expression of ISGs (Rotger and others 2011). Excessive or prolonged IFN-αβ signaling is therefore associated with severe disease in HIV infection. However, host intrinsic defects in pDCs, particularly relating to decreased ability to produce type I IFN, have been associated with enhanced HIV replication (O'Brien and others 2013). Furthermore, HIV, like many other viruses, has inbuilt mechanisms such as the HIV viral protease (Solis and others 2011) dedicated to the antagonism of type I IFNs, and ISGs, including tripartite motif (TRIM)5α, apolipoprotein B mRNA-editing enzyme, catalytic polypeptide-like (APOBEC) 3G, and tetherin, are all known potent antiretroviral factors (reviewed in (Jolly and others 2010; Kirchhoff 2010)). Consequently, HIV must strike a delicate balance where it suppresses IFN-αβ production by infected cells to minimize the antiviral response, yet still stimulate some type I IFN secretion from pDCs to drive inflammation and attract CD4^+^ T cells, thereby creating conditions to favor its own replication.

Similar to previously discussed studies in acute influenza infection, Stary and others (2009) demonstrated in HIV infection that pDC-derived IFN-α led to expression of TRAIL on pDCs and CD4^+^ T cells and concomitant death receptors (TRAIL-R2) on CD4^+^ T cells; this interaction induced apoptosis of uninfected CD4^+^ T cells and consequently severe host pathology. Furthermore, the expression of IFN-α in pDCs and of TRAIL and TRAIL-R2 in tonsil tissue was found to be higher in HIV progressors compared with long-term nonprogressors (Herbeuval and others 2006). It was also shown that HIV and IFN-αβ upregulate proapoptotic molecules, including Bak in T cells, and that in HIV patients, Bak levels in T cells inversely correlated with T cell counts (Fraietta and others 2013). A common theme appears to emerge in both acute and chronic viral infections where type I IFN can mediate immunopathology by driving excessive apoptosis through induction of the TRAIL pathway (Fig. 1).

This is further exemplified by data implicating type I IFN in driving pathogenic innate/adaptive interactions, in particular through the TRAIL pathway, in HBV infection (Dunn and others 2007; Micco and others 2013; Peppa and others 2013). NK cells in the blood and liver of patients with a pathogenic outcome of persistent HBV infection (flares of liver inflammation) were found to have increased activation and TRAIL expression on their circulating and intrahepatic NK cells in association with intermittent induction of IFN-α (Dunn and others 2007). Subsequently, both endogenous and therapeutic IFN-α were shown to expand TRAIL+ NK cells in the context of HCV infection (Ahlenstiel and others 2010; Stegmann and others 2010; Ahlenstiel and others 2011). Administration of therapeutic doses of IFN-α likewise potently induced *in vivo* NK cell activation and TRAIL expression in patients with HBV, with their proliferative expansion being attributed to the induction of IL-15 (Micco and others 2013). TRAIL+ NK cells can drive pathogenesis in viral hepatitis by killing hepatocytes (Dunn and others 2007; Liang and others 2007; Stegmann and others 2010) and by deleting virus-specific T cells that upregulate the TRAIL-R2 receptor in the HBV-infected liver (Peppa and others 2013). NK cells that have been expanded and activated by type I IFN could potentially downregulate antiviral immunity through several additional mechanisms recently defined in mouse models, including cytotoxic killing of APC or T cells and production of IL-10 (Waggoner and others 2012; Crome and others 2013; Crouse and others 2014b). Conversely, T cells, at least in the setting of type I IFN induction in some acute viral infections (LCMV and VSV), may modulate their ligand expression to specifically protect themselves from NK cell killing (Crouse and others 2014a; Xu and others 2014).

The pathogenic effects of type I IFN on adaptive immunity in persistent viral infection are further evidenced by the marked decrease in global T cells noted in patients with chronic HCV or HBV treated with this cytokine (Barnes and others 2009; Micco and others 2013). Virus-specific T cells are not reconstituted in these patients upon IFN-α treatment, even when HBV or HCV infection are resolved (Barnes and others 2009; Missale and others 2012; Penna and others 2012; Micco and others 2013). The detrimental effects of IFN-α are underscored by comparison with the marked reconstitution of antiviral T cells that can be achieved in patients clearing HBV and HCV antigens with the new generations of direct-acting antivirals (Boni and others 2012; Martin and others 2014). The beneficial direct antiviral effects of IFN-α, for example, inducing APOBEC cytidine deaminases able to degrade the stable nuclear episomal form of HBV (Lucifora and others 2014), may therefore be counteracted by its pathogenic effects on adaptive immunity. Similarly, the direct anti-HCV activity of IFN-α can also be negatively affected by pre-existing innate immunity. Although HCV has several mechanisms to downregulate type I IFN induction (inactivating MAVS and TRIF), ISGs are still induced in infected hepatocytes in most chronically infected patients. Paradoxically, HCV patients with high pre-existing levels of certain ISGs in their hepatocytes (likely partially induced by endogenous IFN-λ) are less likely to respond to IFN-α therapy than those with lower levels (Chen and others 2005; Heim and Thimme 2014).

## Bacterial Infection

While type I IFNs have been shown to contribute to protection in many bacterial infections (MacMicking 2012), there is growing evidence that they may impede protection against some intracellular bacteria. Early indications of a disease-promoting effect of type I IFNs came from 3 studies on Listeria infection, showing that *Ifnar1*−/− mice were more resistant to infection than wild-type mice (Auerbuch and others 2004; Carrero and others 2004; O'Connell and others 2004). The proposed mechanisms were type I IFN-induced apoptosis of lymphocytes and type I IFN-induced downregulation of the IFN-γ receptor on macrophages, thus blocking the induction of crucial antibacterial programs by IFN-γ in these cells (Rayamajhi and others 2010; Kearney and others 2013). Similarly, in studies of *Mycobacterium tuberculosis* (*Mtb*) infection in mice, it was shown that more virulent strains induced higher type I IFN levels (Manca and others 2005) and that addition of exogenous type I IFN (Manca and others 2001) or its induction by the TLR3 agonist polyI:C (Antonelli and others 2010) decreased bacterial control and increased susceptibility. The absence of a TPL-2, a negative regulator of type I IFN, led to increased type I IFN levels and loss of *Mtb* control in infected KO mice, and this effect was entirely reversed by crossing *Tpl2*−/− mice to *Ifnar1*−/− mice (McNab and others 2013).

The relevance of murine-derived data from infection models for human tuberculosis became clear when a type I IFN-driven transcriptional signature was found in the blood of patients with active, but not latent, Tb (Berry and others 2010). It was subsequently shown that successful treatment of active Tb leads to the disappearance of this type I IFN signature, thus correlating type I IFN closely with lack of *Mtb* control (Bloom and others 2012). Similar to Listeria infection, IFN-γ-driven Th1 responses are crucial for protection from *Mtb* and therefore one of the suggested mechanisms for type I IFN-mediated loss of *Mtb* control is the blockade of Th1 responses (Manca and others 2001). It is known that type I IFNs induce the immunosuppressive cytokine IL-10 and therefore reduced immune responses due to type I IFN-induced IL-10 contribute to loss of *Mtb* control in infection (McNab and others 2014).

IL-1β is another antibacterial cytokine that protects against *Mtb* and is suppressed by type I IFNs (Mayer-Barber and others 2011; Novikov and others 2011). Reciprocal control of type I IFNs by IL-1β through a prostaglandin E2-mediated mechanism was recently demonstrated, and prostaglandin E2 treatment reduced type I IFN levels and increased protection in *Mtb* infection (Xu and others 2008; Mayer-Barber and others 2014). Similarly, in *Mycobacterium leprae* infections of humans, an IL-10-driven suppression of IFN-γ responses by type I IFNs has been associated with the lepromatous rather than the better controlled tuberculoid form of the disease (Teles and others 2013).

In conclusion, in several bacterial infections requiring strong IFN-γ responses for protection, type I IFN appears to be detrimental, and at least 3 separate, but overlapping, type I IFN-mediated mechanisms are involved: induction of excessive apoptosis, specific suppression of Th1 and IFN-γ responses or of IL-1β required for bacterial control, and dampening of the immune response by strong IL-10 induction.

## Viral–Bacterial Coinfection

The phenomenon of virus infection facilitating secondary bacterial coinfection is probably best described in human outbreaks, and best studied in mouse models, for the combinations of influenza and *Streptococcus pneumoniae* or *Staphylococcus aureus* (McCullers 2014). In both seasonal influenza and in pandemics, a high percentage of patients with influenza are also infected with these bacteria and others such as *Haemophilus influenzae* and *Moraxella*. Already in the 1918 pandemic caused by a highly pathogenic influenza A strain, more than 90% of all biopsies or necropsies showed bacterial superinfection (Morens and others 2008). Given the wealth of evidence demonstrating that high type I IFN levels can impair the antibacterial immune response, it is hardly surprising that in viral–bacterial coinfections, type I IFNs have been proposed as important players that facilitate secondary bacterial infection. In these studies, mechanisms proposed include the suppression of Th17 and neutrophil responses, with reduction of neutrophil generation, recruitment, and survival (Navarini and others 2006), suppression of neutrophil chemoattractants such as KC (Shahangian and others 2009), and reduced IL-17 production by T cells (Kudva and others 2011; Li and others 2012). At least in gamma-delta T cells, this type I IFN-induced reduction in IL-17 production appears to be IL-27 mediated (Cao and others 2014).

The reciprocal regulation between KC and type I IFN was also addressed in a study of the cell types recruited into influenza-infected lungs in wild-type and *Ifnar1*−/− mice. In this study, absence of type I IFN signaling allowed for the accumulation of Ly6C^int^ monocytes producing high amounts of KC, while in wild-type mice, Ly6C^high^ cells producing mainly CCL2 were the predominant monocyte population (Seo and others 2011). In addition to the blockade of Th17 responses by virus-induced type I IFNs, monocyte recruitment has also been shown to be impaired through suppression of the chemoattractant CCL2 (Nakamura and others 2011), findings that are somewhat discrepant to those by Seo and others (2011). These differences may be due to the specific model of bacterial colonization of the upper respiratory tract used by Nakamura and others (2011). In this model, high-density bacterial colonization and invasion are promoted by viral infection. Type I IFN-mediated loss of Mtb control was also shown in influenza–*Mtb* coinfection (Redford and others 2014).

Hence, it appears that type I IFNs can reduce both Th1 and Th17 responses and therefore impair responses to both intracellular and extracellular bacteria. Only a few studies have so far attempted to identify the molecular mechanism underlying such IFN-αβ-mediated cytokine modifications. Schliehe and others (2015) have recently shown that the histone methyltransferase Setdb2 is induced by IFN-αβ and imprints a repressive histone mark on the Cxcl1 promotors. In influenza–bacterial coinfection, this results in reduced CXCL1 secretion, less neutrophil recruitment, and reduced bacterial control, thus identifying a molecular pathway leading from influenza-induced IFN-αβ to facilitated bacterial invasion. More studies like this will pave the way to a molecular understanding of the predisposition to viral–bacterial coinfection and how type I IFN signaling contributes to this.

## Conclusions

We have come a long way in IFN research from the initial focus on antiviral effects—these are important, but many IFN-αβ-driven processes, including immune modulation, apoptosis induction, and proliferative blockade, happen in parallel. The net effect that type IFNs have during infections will depend on how these different actions are balanced, helping to explain why IFN-αβ as a treatment option in infectious diseases has proved less straight forward than might originally have been assumed.

The divergent results obtained by using *Ifnar1*−/− mice and by exogenous addition of type I IFNs in viral infections serve as a good example to demonstrate the pleiotropic functions of the type I IFN family. Like many other biological effects, the outcome of type I IFN signaling is a bell-shaped curve (Fig. 2). A low concentration will alert a cell to viral infection and allow for induction of antiviral genes; a bit more will indicate to a given cell that viral infection has taken hold and it is time to recruit immune cells to aid in viral clearance or prime an infected cell for apoptosis. Signaling beyond this drives overactivation of the immune response and leads to tissue damage through inducing apoptosis or blocking proliferation of tissue cells. To control for this, the organism has developed many mechanisms that are mostly, but not always, fail-safe. Type III IFNs are an elegant additional system, which allows for activation of the antiviral state without activating the immune system. While tight regulation of IFN-αβ expression is required to restrict the otherwise global effects of this cytokine, type III IFNs may need less regulation as receptor expression is constitutively restricted. Understanding better the complex interplays between the IFN systems might allow us to one day use these potent cytokines in a more focused and therefore more successful manner.
